# The Potential Adverse Impact of Post-Treatment Thrombocytopenia on Clinical Outcomes in Cancer Patients Treated With Immune Checkpoint Inhibitors

**DOI:** 10.7759/cureus.62163

**Published:** 2024-06-11

**Authors:** Asım Armağan Aydın, Füsun Topçugil

**Affiliations:** 1 Department of Medical Oncology, Antalya Education and Research Hospital, Antalya, TUR; 2 Department of Internal Medicine, Batıgöz Health Group Private Hospital, İzmir, TUR

**Keywords:** immune checkpoint inhibitor, survival, immunotherapy, cancer, immune-related adverse events, thrombocytopenia

## Abstract

Background: The main goal of this study is to explore the prognostic and predictive implications of post-treatment thrombocytopenia on treatment efficacy and clinical outcomes in advanced-stage cancer treated with immune checkpoint inhibitors (ICIs).

Methods: This retrospective study included 102 patients with advanced-stage cancer who were treated with ICIs. The simultaneous administration of chemotherapy and ICIs was omitted; nevertheless, the selection of chemotherapy agents employed in different treatment lines was left to the discretion of the attending clinician. Patients were stratified into distinct cohorts based on their post-treatment platelet counts (evaluated for up to four to six months after the completion of ICI). The primary endpoint of interest was progression-free survival (PFS), and overall survival (OS) was the secondary endpoint.

Results: Patients with superior Eastern Cooperative Oncology Group (ECOG) performance status and those who received ICI as second-line treatment displayed markedly elevated incidences of grade 1 thrombocytopenia (p < 0.05). Kaplan-Meier survival analysis confirmed that patients with high-grade thrombocytopenia had significantly shorter PFS (six vs. 13 vs. 19 months, p < 0.0001) and OS (10 vs. 21 vs. 25 months, p < 0.0001) than those with lower grades or without thrombocytopenia, respectively. Multivariate analysis revealed that decreased platelet levels were a negative independent prognostic factor for both PFS and OS in patients with advanced-stage cancer who received ICIs.

Conclusion: The results of this retrospective study suggest that a decline in platelet levels after treatment represents a dependable adverse prognostic biomarker for clinical outcomes. Moreover, a decrease in platelet levels has been linked to reduced treatment efficacy in advanced-stage cancer patients receiving ICIs, thereby providing valuable prognostic insights for the implementation of personalized treatment strategies in cancer immunotherapy.

## Introduction

Following research conducted in the last decade, immune check point inhibitors (ICIs) have become the cornerstone of standard treatment for various types of cancer [1]. Following the Food and Drug Administration’s (FDA) approval of nivolumab, a PD-1 inhibitor, for the treatment of advanced-stage melanoma in 2015 [2], there has been a notable increase in research efforts directed toward therapies targeting checkpoint inhibitors, such as CTLA-4, PD-1, and its associated ligand PD-L1. ICIs target these checkpoints, thereby enabling the immune system to identify and combat cancer cells.

Despite their increased utilization in cancer therapy and superior tolerability compared to cytotoxic chemotherapy regimens, ICIs have also been linked to a spectrum of toxicities known as immune-related adverse events (irAEs) [3]. Research examining the correlation between irAEs and treatment effectiveness, as well as clinical outcomes, is gaining traction in scholarly literature. The most frequently encountered irAEs were gastrointestinal, endocrine, and dermatological toxicity. In a study carried out by Cortellini et al., findings revealed a correlation between heightened irAEs and improved survival outcomes in non-small cell lung cancer (NSCLC) treated with ICIs [4]. In a study by Ng et al., focusing on patients with hepatocellular carcinoma (HCC) undergoing treatment with ICIs, a similar trend was observed, indicating that escalating occurrences of severe irAEs correlated with enhanced prognostic outcomes [5]. In addition, a meta-analysis conducted by Fan et al. revealed that the development of irAEs predicted superior tumor responses and increased survival rates among patients with diverse cancer types receiving ICIs [6].

Although hematological system-related adverse effects are relatively less frequent (such as autoimmune hemolytic anemia and immune thrombocytopenia), they can lead to challenges in treatment management, thereby negatively impacting clinical outcomes [7]. ICI-related thrombocytopenia (irTP) is characterized by accelerated autoimmune destruction of platelets due to increased levels of cytokines related to T-cell proliferation, decreased platelet production as a result of impairment in megakaryopoiesis, and increased platelet consumption due to the activation of the coagulation cascade associated with immune-mediated endothelial damage [8]. The management of irTP involves a close monitoring of platelet counts and prompt intervention with corticosteroids, intravenous immunoglobulins, or other immunosuppressive agents, if severe thrombocytopenia occurs [9,10]. Understanding the underlying mechanisms of irTP is crucial for developing strategies to prevent, diagnose, and manage this adverse event in cancer patients receiving ICI therapy [10].

Regrettably, despite their association with prolonged responses and sustained remission in numerous cases, the effectiveness of ICIs remains limited within certain patient subgroups. In addition, the fundamental mechanisms involved in identifying specific clinical, cellular, and molecular predictive biomarkers remain unclear. Based on this premise, the objective of our study was to scrutinize the predictive capacity of post-treatment fluctuations in platelet levels and their influence on response rates and clinical outcomes in a cohort of patients undergoing ICI therapy for advanced-stage cancer. This endeavor aimed to enhance the therapeutic efficacy of ICIs by identifying cellular and molecular biomarkers with prognostic capabilities, enabling the differentiation of patient cohorts poised to derive maximal benefit. Moreover, it seeks to facilitate the precise selection of personalized and targeted therapeutic modalities with increased accuracy.

## Materials and methods

Study design

Between April 2019 and March 2024, patients aged ≥18 years who underwent treatment with ICIs for various metastatic malignancies at the Oncology Department of the Health Science University Antalya Education and Research Hospital (HSUAERH) in Antalya, Turkey, were retrospectively evaluated. Patients did not receive concurrent chemotherapy with ICI; however, the selection of chemotherapy agents administered at different lines was at the discretion of the clinician. The demographic and clinical profiles of the patients included variables, such as age, sex, cancer type, Eastern Cooperative Oncology Group (ECOG) performance status, body mass index, smoking status, presence of comorbidities, specific ICI utilized, and line of ICI. Pre-treatment platelet counts (within 10 days preceding the initiation of ICI therapy) and post-treatment platelet counts (evaluated for up to four to six months after the completion of ICI treatment) were obtained.

According to the guideline "Management of Immune-Related Adverse Events in Patients Treated with Immune Checkpoint Inhibitor Therapy" published by the American Society of Clinical Oncology [9], patients were stratified into distinct categories based on their post-treatment platelet levels: Group 1 (with a normal platelet count of ≥150,000); Group 2 (comprising individuals with grade 1 thrombocytopenia, characterized by platelet counts ranging from 75,000 to 150,000); and Group 3 (encompassing patients with grades 2-4 thrombocytopenia, defined by platelet counts <75,000) (Table 1).

**Table 1:** Classification based on the pre-treatment and post-treatment platelet levels for all patients.

Variable	All patients (n = 102)
Baseline platelet count (x10^3^/µl), median (range)	380 (165-603)
Nadir platelet count (x10^3^/µl), median (range)	203 (10-424)
Group 1 ≥150,000 (x10^3^/µl) (No thrombocytopenia), n(%)	74 (72.5)
Group 2 75,000-150,000 (x10^3^/µl) (Grade 1 thrombocytopenia),n(%)	23 (22.6)
Group 3 <75,000 (x10^3^/µl) (Grade 2-4 thrombocytopenia), n(%)	5 (4.9)
Time to nadir platelet count (days), median (range)	58 (12-176)

Clinical responses were assessed and categorized as complete response (CR), partial response (PR), stable disease (SD), or progressive disease (PD), in accordance with the revised Response Evaluation Criteria in Solid Tumors (RECIST) guidelines (version 1.1). Progression-free survival (PFS) was defined as the time from the initiation of ICIs to progression, death, or last follow-up. Overall survival (OS) was calculated as the time from the initiation of ICIs to death or the last follow-up. The primary endpoint of interest was PFS, whereas OS was the secondary endpoint.

The exclusion criteria included a history of autoimmune disease, use of steroids or other immunosuppressive therapies, and a history of receiving platelet-containing blood transfusions within 90 days prior to ICI treatment. A total of 138 patients were screened. Nine patients with platelet values <150,000 at diagnosis, eight patients with missing clinical data or lost to follow-up and 19 patients with a history of steroid and/or antibiotic use were excluded from the study. Ultimately, 102 patients meeting the inclusion criteria were enrolled in the study for final analysis.

Ethical considerations were adhered to throughout this study, which was conducted in compliance with the Helsinki Declaration of 1964 as revised in 2008. The study protocol was thoroughly reviewed and approved by the Institutional Review Board of the HSUAERH (approval number: 2023/345).

Statistical analysis

Statistical analyses were conducted using the Statistical Package for Social Sciences (SPSS) for Windows version 27 (IBM SPSS Inc., Chicago, IL, USA). The normality of continuous data distribution was evaluated using either the Kolmogorov-Smirnov test or the Shapiro-Wilk test. Continuous data were analyzed using the independent sample Mann-Whitney U test. Categorical data were assessed using Pearson's chi-square test and Fisher's exact test. OS and PFS were calculated using the Kaplan-Meier method, and survival among the study groups was compared using the log-rank test. Statistical significance was defined as p < 0.05.

## Results

A total of 138 patients with metastatic cancer treated with ICIs were enrolled in this study. Nine patients with platelet values <150,000 at diagnosis, 19 patients with a history of steroid and/or antibiotic use, and eight patients for whom data could not be obtained during clinical follow-up were excluded from the study. The median age at diagnosis was 63.6 ± 9.6 years (range: 41-83), and 77.5% (n = 77) were male. While 45.1% of the patients smoked, 33.3% had at least one comorbid condition. The most common cancer diagnoses were NSCLC (60.8%), genitourinary cancer (14.7%), and melanoma (13.7%). Nivolumab was the most commonly used ICI (86.3%). Some of the patients (22.5%) received radiotherapy at any stage of ICI treatment.

In patients receiving ICI therapy, the median number of treatment cycles was 14 (range: 2-61). The median baseline platelet count before ICI therapy and post-treatment nadir platelet count were 380,000 and 203,000, respectively. The mean duration until the post-treatment nadir platelet count was 58 days. Most patients (70.6%) received at least one line of therapy before ICI initiation. None of the patients received platelet transfusions within 90 days of ICI initiation. A complete response was observed in 8.8% of patients, while a partial response was observed in 42.2% of patients. The demographic and clinical data of the patients are summarized in Table 2.

**Table 2:** Distribution of demographic and clinical data among the patient population. Abbreviations: ECOG PS, Eastern Cooperative Oncology Group performance status; ICI, immune checkpoint inhibitor; NSCLC, non-small cell lung cancer; SCLC, small-cell lung cancer; PD, progressive disease; SD, stable disease; PR, partial remission; CR, complete remission

Variables		n	%
Age	<65	51	50.0%
	≥65	51	50.0%
Sex	Male	77	75.5%
	Female	25	24.5%
Comorbidity	Absent	68	66.7%
	Present	34	33.3%
Smoking status	Absent	56	54.9%
	Present	46	45.1%
Cancer type	NSCLC	62	60.8%
	SCLC	3	2.9%
	Melanoma	14	13.7%
	Genitourinary	15	14.7%
	Hepatobilliary	4	3.9%
	Gynecologic	2	2.0%
	Others	2	2.0%
ECOG PS	0-1	69	67.6%
	2	33	32.4%
Checkpoint inhibitor	Nivolumab	88	86.3%
	Atezolizumab	6	5.9%
	Pembrolizumab	5	4.9%
	Durvalumab	3	2.9%
Line of ICI	1	30	29.4%
	2	63	61.8%
	≥3	9	8.8%
Radiotherapy	Absent	79	77.5%
	Present	23	22.5%
Treatment response	PD	18	17.6%
	SD	32	31.4%
	PR	43	42.2%
	CR	9	8.8%
Post-treatment thrombocytopenia	Absent	74	72.5%
	Present	28	27.5%
Groups classified based on post-treatment platelet levels	Group 1 (no thrombocytopenia)	74	72.5%
	Group 2 (Grade 1)	23	22.6%
	Group 3 (Grade 2-4)	5	4.9%
Progression	Absent	25	24.5%
	Present	77	75.5%
Exitus	Absent	42	41.2%
	Present	60	58.8%

During the mean follow-up duration of 20.1 months, progression occurred in 77 patients (77.5%) and death occurred in 60 patients (%58.8). The median PFS and OS were 9 (95% confidence interval (CI): 5.9-12.1) and 14 months (95% CI: 10.8-17.2), respectively. In 74 patients (72.5%), thrombocytopenia did not develop, while grade 1 thrombocytopenia was observed in 23 patients (22.6%), and grade 2-4 thrombocytopenia was observed in five patients (4.9%). Patients with superior ECOG PS and those who received ICI as second-line treatment displayed markedly elevated incidences of grade 1 thrombocytopenia (p < 0.05). Furthermore, the group experiencing grade 1 thrombocytopenia exhibited notably heightened objective response rates, reaching 95% (p < 0.05). The relationships between groups classified according to post-treatment platelet levels and clinical characteristics are summarized in Table 3.

**Table 3:** Relationship between groups classified according to post-treatment platelet levels and clinical characteristics. Abbreviations: ECOG PS, Eastern Cooperative Oncology Group performance status; ICI, immune check-point inhibitor; NSCLC, non-small cell lung cancer; SCLC, small-cell lung cancer; PD, progressive disease; SD, stable disease; PR, partial remission; CR, complete remission *Chi-square test, p < 0.05 is significant.

Variables		Post-treatment thrombocytopenia			
		No thrombocytopenia	Grade 1	Grades 2-4	p
		n (%)	n (%)	n (%)	
Age	<65	37 (50%)	13 (56.5%)	1 (20%)	0,6*
	≥65	37 (50%)	10 (43.5%)	4 (80%)	
Sex	Male	57 (77%)	17 (73.9%)	3 (60%)	0,437*
	Female	17 (23%)	6 (26.1%)	2 (40%)	
Comorbidity	Absent	50 (67.6%)	15 (65.2%)	3 (60%)	0,71*
	Present	24 (32.4%)	8 (34.8%)	2 (40%)	
Smoking	Absent	40 (54.1%)	13 (56.5%)	3 (60%)	0,756*
	Present	34 (45.9%)	10 (43.5%)	2 (40%)	
ECOG PS	0-1	44 (59.5%)	22 (95.7%)	3 (60%)	0,034*
	2	30 (40.5%)	1 (4.3%)	2 (40%)	
Cancer type	NSCLC	48 (64.9%)	13 (56.5%)	1 (20%)	0,569*
	SCLC	0 (0%)	1 (4.3%)	2 (40%)	
	Melanoma	10 (13.5%)	3 (13%)	1 (20%)	
	Genitourinary	10 (13.5%)	5 (21.7%)	0 (0%)	
	Hepatobilliary	2 (2.7%)	1 (4.3%)	1 (20%)	
	Gynecologic	2 (2.7%)	0 (0%)	0 (0%)	
	Diğer	2 (2.7%)	0 (0%)	0 (0%)	
Checkpoint inhibitor	Nivolumab	67 (90.5%)	19 (82.6%)	2 (40%)	0,041*
	Atezolizumab	1 (1.4%)	3 (13%)	2 (40%)	
	Pembrolizumab	5 (6.8%)	0 (0%)	0 (0%)	
	Durvalumab	1 (1.4%)	1 (4.3%)	1 (20%)	
Line of ICI	1	19 (25.7%)	7 (30.4%)	4 (80%)	0,014*
	2	46 (62.2%)	16 (69.6%)	1 (20%)	
	≥3	9 (12.2%)	0 (0%)	0 (0%)	
Radiotherapy	Absent	59 (79.7%)	17 (73.9%)	3 (60%)	0,284*
	Present	15 (20.3%)	6 (26.1%)	2 (40%)	
Treatment response	PD	17 (23.0%)	1 (4.3%)	0 (0%)	<0,001*
	SD	29 (39.2%)	2 (8.7%)	1 (20%)	
	PR	28 (37.8%)	12 (52.2%)	3 (60%)	
	CR	0 (0%)	8 (34.8%)	1 (20%)	
Post-treatment thrombocytopenia	Absent	74 (100%)	0 (0%)	0 (0%)	<0,001*
	Present	0 (0%)	23 (100%)	5 (100%)	

Survival analyses among groups classified according to post-treatment platelet levels

**Figure 1:**
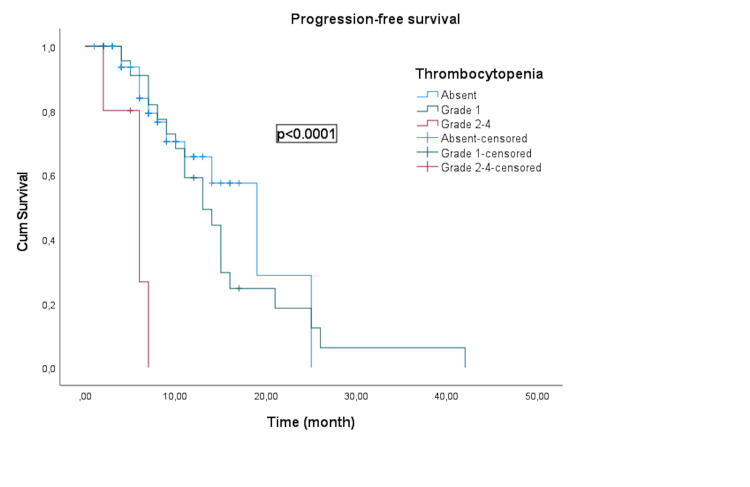
Kaplan-Meier curve illustrating the progression-free survival of groups according to post-treatment platelet levels.

The median PFS for patients without thrombocytopenia, grade 1 thrombocytopenia, and grades 2-4 thrombocytopenia was 19 months (95% CI: 11.9-26.2), 13 months (95% CI: 8.7-17.3), and six months (95% CI: 2.7-9.3), respectively (Figure 1 ).

**Figure 2:**
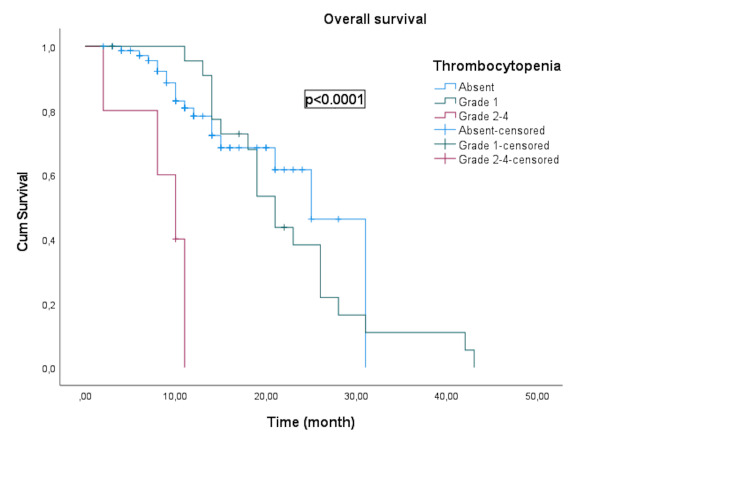
Kaplan-Meier curve illustrating the overall survival of groups according to post-treatment platelet levels.

The median OS for patients without thrombocytopenia, grade 1 thrombocytopenia, and grade 2-4 thrombocytopenia was 25 months (95% CI: 20.2-29.8), 21 months (95% CI: 18.4-23.6), and 10 months (95% CI: 5.7-14.3), respectively (Figure 2).

Patients with high-grade thrombocytopenia (grades 2-4) demonstrated notably shorter PFS (p < 0.0001) and OS (p < 0.0001) than those with lower grades or without thrombocytopenia.

## Discussion

The relationship between emerging side effects during cancer treatment and treatment efficacy has been demonstrated in previous studies, particularly in the field of molecular and targeted therapies for cancer treatment [11]. Recently, owing to their biological structure, potent efficacy, and tolerability advantages, the use of ICIs in this area has become widespread, leading to an acceleration in studies focusing on the prognostic predictive outcomes of findings termed as immune-related adverse events [5,6]. While many of these studies have predominantly focused on dermatological, endocrine, and gastrointestinal side effects, research on hematological side effects, although relatively uncommon, is also emerging. In a study by Lee et al., increased lymphocyte levels following ICI therapy in NSCLC were associated with more favorable PFS and OS outcomes [12].

Platelets engage in reciprocal interactions with tumor cells, fostering their survival, proliferation, and metastatic potential [13]. They create a defensive barrier around circulating tumor cells, shielding them from immune surveillance and facilitating their dissemination to distant anatomical sites [14]. Platelets release an array of growth factors and cytokines, notably vascular endothelial growth factor (VEGF), which orchestrates angiogenesis and metastasis. Platelets attenuate their antitumor efficacy by modulating the activity of immune effectors, such as T cells and natural killer cells. In addition, platelets facilitate the extravasation of tumor cells from circulation into the surrounding tissues, augment their adhesion to endothelial surfaces, and enhance their invasive capacity. Tumor-derived factors, such as thrombopoietin, stimulate platelet production in the bone marrow. Thrombocytosis correlates with unfavorable prognoses across diverse cancer types, exacerbating disease progression, and metastasis. Another hypothesis posits that certain tumor-associated moieties induce phenotypic alterations in platelets, giving rise to the so-called tumor-educated platelets [15]. The interplay between cancer cells and primed activated platelets results in nuanced cellular dynamics. Recent investigations have revealed the expression of programmed death-ligand 1 (PDL-1) in platelets, with expression profiles differing in patients undergoing ICI therapy, albeit lacking significant effects on the total platelet count [16]. These observations underscore the intricate and multifaceted nature of the interplay between cancer cells and platelets and offer promising avenues for therapeutic interventions. However, further elucidation of the intricate interplay between cancer biology and platelet physiology is needed.

Our investigation supports the notion that reduction in platelet counts after treatment with ICIs is significantly correlated with shorter PFS and OS. A study conducted by Assi et al. revealed that the development of post-treatment thrombocytopenia was associated with extended OS in cancer patients treated with ICIs [17]. The results did not reflect the PFS outcomes. By contrast, findings from a study by Haddad et al. illustrated a comparable correlation between diminished platelet levels and unfavorable OS outcomes in patients undergoing combined ICI and cytotoxic chemotherapy [18]. Combined with the data obtained from our investigation, it can be inferred that irTP is a promising negative predictive indicator of treatment efficacy in patients receiving ICIs, offering valuable prognostic insights for the refined implementation of personalized therapeutic strategies in cancer immunotherapy.

This study has several limitations. The retrospective design and narrow cohort made it difficult to interpret the linear relationship between thrombocytopenia and treatment efficacy. Factors such as variations in ICI treatment agents, administration of ICIs at different lines, and the presence of cancer types with diverse clinicopathological characteristics can also be considered disadvantages of the study. Conditions that can affect the timing of response assessment to ICI treatment, such as pseudoprogression and organ dysfunction that can develop after cytotoxic chemotherapies given in previous lines, indirectly affect OS due to decreased treatment tolerability. The small number of cases with high-grade (grade ≥2) thrombocytopenia made the statistical interpretation challenging in this group. Therefore, further confirmation of these findings is required through prospective studies with larger cohorts. In addition to these and similar studies, the role of platelets in cancer immunology has become more prominent. Thus, efforts should be made to conduct comprehensive research on predictive and prognostic biomarkers to optimize personalized treatments for cancer immunotherapy.

## Conclusions

Platelets are becoming increasingly prominent in cancer immunotherapy owing to their physiological and structural characteristics. IrTP may serve as an effective prognostic tool for assessing responses to ICIs. Future studies focusing on predictive and prognostic biomarkers that better reflect the foundation of immunological treatment are required to facilitate the interpretation of treatment efficacy and clinical outcomes, thereby achieving optimal treatment success in cancer immunotherapy.
